# The Multi-Omics Architecture of Juvenile Idiopathic Arthritis

**DOI:** 10.3390/cells9102301

**Published:** 2020-10-15

**Authors:** Xiaoyuan Hou, Huiqi Qu, Sipeng Zhang, Xiaohui Qi, Hakon Hakonarson, Qianghua Xia, Jin Li

**Affiliations:** 1 Department of Cell Biology, the Province and Ministry Co-sponsored Collaborative Innovation Center for Medical Epigenetics, School of Basic Medical Sciences, Tianjin Medical University, Tianjin 300070, China; houxiaoyuan@tmu.edu.cn (X.H.); zsp@tmu.edu.cn (S.Z.); qixiaohui@tmu.edu.cn (X.Q.); 2 Center for Applied Genomics, the Children’s Hospital of Philadelphia, Philadelphia, PA 19104, USA; quh@email.chop.edu (H.Q.); hakonarson@email.chop.edu (H.H.); 3 Division of Human Genetics, Children’s Hospital of Philadelphia, Philadelphia, PA 19104, USA; 4 Department of Pediatrics, the Perelman School of Medicine, University of Pennsylvania, Philadelphia, PA 19104, USA; 5 Tianjin Eye Hospital, Tianjin 300020, China; 6 Tianjin Key Laboratory of Ophthalmology and Visual Science, Tianjin Eye Institute, Tianjin 300020, China

**Keywords:** epigenetics, genome-wide association study, genetics, juvenile idiopathic arthritis, transcriptome

## Abstract

Juvenile idiopathic arthritis (JIA) is highly heterogeneous in terms of etiology and clinical presentation with ambiguity in JIA classification. The advance of high-throughput omics technologies in recent years has gained us significant knowledge about the molecular mechanisms of JIA. Besides a minor proportion of JIA cases as monogenic, most JIA cases are polygenic disease caused by autoimmune mechanisms. A number of *HLA* alleles (including both *HLA* class I and class II genes), and 23 non-*HLA* genetic loci have been identified of association with different JIA subtypes. Omics technologies, i.e., transcriptome profiling and epigenomic analysis, contributed significant knowledge on the molecular mechanisms of JIA in addition to the genetic approach. New molecular knowledge on different JIA subtypes enables us to reconsider the JIA classification, but also highlights novel therapeutic targets to develop a cure for the devastating JIA.

## 1. Introduction

Juvenile idiopathic arthritis (JIA) is a common type of chronic rheumatic diseases affecting children with the age of onset under 16 years, and an important cause of disability. Epidemiological studies showed that incidence rate of JIA ranges from 1.6 to 23 per 100,000 children annually and the prevalence of JIA was about 3.8–400 per 100,000 children in Europe [1]. Girls exhibit a higher incidence rate than boys (10.0/100,000 compared to 5.7/100,000) [1]. Its negative effects on children’s physical development, as well as psychiatric development, cause serious damage to the quality of life of affected children, causing pain of active joints, physical disability, anxiety, and depression. There is no cure for JIA. JIA needs aggressive treatment to control its symptoms. For this difficult disease, it is important to understand the underlying molecular mechanisms of the development of JIA systematically through unbiased approaches. In recent years, people have started to gain knowledge about the multiomics architecture of JIA, thanks to the advance of high-throughput omics technologies, which is the focus of this review article.

## 2. JIA as a Heterogeneous Group of Diseases

JIA is a group of diseases, highly heterogeneous in terms of etiology and clinical presentation. It is classified into seven subtypes according to the Pediatric Task Force of the International League of Associations for Rheumatology (ILAR) [2], including systemic arthritis, oligoarthritis, polyarthritis RF-negative, polyarthritis RF-positive, psoriatic arthritis, enthesitis-related arthritis, and undifferentiated arthritis [2]. In terms of the seven subtypes of JIA, oligoarthritis is the largest category of JIA, accounting for 50–60% of all cases; the other subtypes’ frequencies are, polyarthritis (30–35%), systemic JIA (10–20%), psoriatic arthritis (2–15%), enthesitis-related arthritis (1–7%) [3]. After 25 years since the clinical application of the ILAR classification, it is understood, to date, that some subtypes of JIA are highly heterogeneous, e.g., the polyarthritis RF-negative and psoriatic subtypes [4]. Ambiguity in classifying certain patients has also been an issue. Clinical efforts are currently being made to revise the ILAR classification [5].

### 2.1. Systemic JIA (sJIA)

Systemic JIA (sJIA) is defined as fever and arthritis that last for 2 weeks or more, with one or more of the following features: (1) transient erythema, (2) lymphadenopathy, (3) hepatomegaly or splenomegaly, and (4) serositis [6]. However, sJIA is often difficult to diagnose because its symptoms are nonspecific, and highly similar to other inflammatory diseases [7]. Due to the significant similarity with adult Still’s disease, it seems correct to regard sJIA as juvenile onset Still’s disease [4]. As a typical autoinflammatory disease, sJIA is considered as a polygenic disease as adult Still’s disease [8], rather than a monogenic disease. Although commonly with fever and joint symptoms, almost all autoinflammatory diseases with a monogenic etiology, such as TNF receptor-associated periodic fever syndrome and juvenile sarcoidosis/Blau syndrome, have a narrowly defined spontaneous feature and systemic inflammation [9].

### 2.2. Oligoarthritis and Polyarthritis

The JIA subtypes, oligoarthritis and polyarthritis, are differentiated only by the number of affected joints. Oligoarthritis affects four or fewer joints during the first 6 months after the onset of the disease. The oligoarticular subtype of JIA is further divided into two subtypes: persistent oligoarthritis and extended oligoarthritis. For the former, the number of joints involved is limited to four or less during the whole disease process, while for the latter, the number of joints involved is more than four after 6 months of onset. Compared to oligoarthritis, polyarthritis affects five or more joints in the first 6 months of disease onset. According to the presence or absence of rheumatoid factor (RF), it is further divided into subtypes of RF positive and RF negative [6]. There are a lot of features in common between oligoarticular and polyarticular JIA, such as relatively good reactivity and benign prognosis [10].

### 2.3. Psoriatic Arthritis

Psoriatic arthritis is characterized by typical psoriatic rash or strong family history (first-degree relative) [2].

### 2.4. Enthesitis-Related JIA

Enthesitis-related JIA is defined as an arthritis or inflammation of the attachment point of tendons or ligaments, with at least two of the following symptoms: (1) sacroiliac tenderness or inflammatory lumbosacral and spinal pain, not limited to cervical; (2) HLA-B27 positive; (3) male children with symptoms older than eight years of age. (4) HLA-B27 related diseases in first degree relatives in family history [2,6].

Undifferentiated arthritis, i.e., JIA patients cannot be assigned to one of the above JIA subtypes or can be assigned to more than one JIA subtype [2].

## 3. Genetic Studies of Monogenic Forms of JIA

Considering the poorly known etiologies of JIA and ambiguities of JIA classification, identification of monogenic forms of JIA has gained us significant knowledge on some critical molecular mechanisms (Table 1).

### 3.1. LACC1

FAMIN, encoded by the laccase domain containing 1 gene (*LACC1*), is an important protein in mitochondrial energy metabolism, related to the NOD2 pathway [11]. Recently, studies found that recessive hereditary *LACC1/FAMIN* mutations were correlated with different subtypes of monogenic JIA, including sJIA [12], oligoarthritis [12], polyarthritis RF-negative [13], and enthesitis-related arthritis [14]. The study using a combination of linkage analysis, homozygosity mapping, and whole-exome sequencing, discovered a homozygous mutation p.Cys284Arg (C284R) of *LACC1* in every affected case of sJIA in five consanguineous families [15]. Besides C284R, the p.Ile254Val (I254V) mutation with important impact on FAMIN function [16], was implicated in nonsystemic forms of JIA [17]. Kallinich et al. found a novel homozygous frameshift mutation (p.T276fs*2) in two siblings with severe oligoarthritis [12]. The study on a Moroccan family with severe arthritis that presented recessive inheritance showed that the frame shift mutation of *LACC1/FAMIN*, p.Cys43Tyrfs*6, was most likely the cause of polyarthritis RF-negative JIA [13].

In a recent in vivo functional study on *LACC1*, Skon-Hegg et al. demonstrated the important role of *LACC1* in inflammatory responses [18]. As shown in this study, *LACC1* knockout mice showed worse disease in the *Citrobacter rodentium* model of colitis, and the collagen-induced arthritis models, compared to wild type (WT) mice, while there were no differences identified between *LACC1* KO and WT in the dextran sulfate sodium (DSS) model of colitis and the K/BxN model of arthritis [18]. Besides JIA, the pathogenic effects of *LACC1* mutation I254V have been correlated with other diseases, e.g., inflammatory bowel disease [19], leprosy [20], and Behçet disease [21]. C284R was also identified of correlation with severe pediatric Crohn’s disease in a Saudi family [15].

### 3.2. LRBA

The LPS responsive beige-like anchor protein gene (*LRBA*) encodes a member of the WDL-BEACH-WD (WBW) gene family, with its expression induced in B cells and macrophages by bacterial LPS [22]. LRBA protein helps to keep intracellular storage of cytotoxic T-lymphocyte-associated antigen 4 (CTLA-4) protein and prevents its degradation [23]. For activation of T lymphocytes, the T cell receptor (TCR) complex requires the costimulation by CD28 after antigen recognition [24]. CTLA-4 transmits inhibitory signals to attenuate T cell activation by competing for the B7 ligands with its homologue CD28 [25,26]. Reduced CTLA-4 activity is likely associated with JIA [27]. Meanwhile the expression of CTLA-4 in CD4+CD28- T-cells in JIA patients is increased in JIA patients and CD28-T cells are not susceptible to inhibition by CTLA-4 [28]. *LRBA* deficiency is a cause of common variable immunodeficiency (CVID), a group of congenital dysregulatory disorders in immune system. According to the study by Azizi et al., 10.1% of 227 CVID patients show rheumatologic disorders, and JIA is the most common manifestation (3.1%) [29]. Lopez-Herrera et al. reported for the first time that homozygous mutations of *LRBA* correlated with common variable immunodeficiency-8 with autoimmunity (CVID8) in four consanguineous families with CVID8 [30]. Semo Oz et al. reported a case of *LRBA* deficiency who initially presented as polyarthritis and was diagnosed with JIA [31].

Gámez-Díaz et al. observed that *Lrba* knockout (*Lrba*^−/−^) mice showed reduced *CTLA4* expression by Tregs and activated conventional CD4+ and CD8+ T lymphocytes, decreased frequency of peritoneal B-1a cells with reduced IL-10 production [32]. Lymphatic tissue showed no obvious signs of autoimmunity. The *Lrba*^−/−^mice can produce a normal amount of serum IgM and IgG, generate specific antibody responses after immunization, and produce elevated serum and secretory basal IgA levels [32]. These findings expanded the roles of *LRBA* protein in dysfunction of immune system, and suggested that *LRBA* might be important for maintaining autoantigen tolerance.

### 3.3. NFIL3

The nuclear factor, interleukin 3 regulated gene (*NFIL3*) encodes an important transcription factor in human immune system, regulating cytokine production of type 2 T helper (T(H)2) cells [33]. Research found that the expression of *NFIL3* is impaired in Crohn’s disease and ulcerative colitis patients, which may shift tolerogenic functions of macrophages to proinflammatory [34]. Schlenner et al. studied two monozygotic twin girls with JIA, using whole exome sequencing, single cell sequencing, and flow cytometry [35]. A novel homozygous mutation, methionine to isoleucine mutation at residue 170 (M170I) in *NFIL3*, was identified in the patients [35]. The mutation reduced stability of *NFIL3* protein. *Nfil3*-knockout mice had upregulated IL-1β production, and increased susceptibility to arthritis induction [35].

### 3.4. UNC13D

The unc-13 homolog D gene (*UNC13D*) encodes a protein essential for cytolytic granules secretion at the immunologic synapse [36]. Partial knockdown expression of *UNC13D* resulted in impaired NK cell degranulation [37]. Schulert et al. reported an intronic mutation in UNC13D, c.117 + 143A > G in a patient with sJIA and recurrent MAS [37]. This mutation is located in a region regulating lymphocyte-specific *UNC13D* expression, disrupted the binding of NF-κB with a transcriptional enhancer, and is associated with downregulated transcription of *UNC13D* in PBMCs. A previous study also reported genetic association between the *MUNC13-4* (*UNC13D*) sequence polymorphisms and sJIA/macrophage activation syndrome (MAS) [38]. Hazen et al. reported compound heterozygous mutations of *UNC13D* correlated with systemic JIA without MAS in an 8-year-old girl with decreased cytotoxic function of NK cells [39].

## 4. Genetic Studies of Polygenic Forms of JIA

Besides the above monogenic forms of JIAs, JIA is commonly considered as an immune disorder caused by complex interactions between environmental factors and multiple genetic risk factors [40]. Early studies revealed that siblings or twins of JIA patients have an increased risk up to 15–30%, which is close to that of type 1 diabetes [41]. The age of onset and disease duration among JIA twins are highly consistent, suggesting JIA as a polygenic genetic disease [40].

In recent years, numerous susceptibility loci have been identified through genome-wide association studies (GWAS) of autoimmune diseases. With the application of molecular genetic and genomic technologies in JIA research, some disease-causing genetic variants have been identified in JIA families [42]. Although some risk variants are located in coding regions of genes affecting protein structure and leading to prominent clinical significance, a larger number of risk variants are mapped to noncoding regions containing regulatory elements, which may have an impact on chromatin structure or noncoding RNA binding, and affect gene expression in a tissue-specific or disease state-related manner [42]. The functional genes in these loci have significantly increased the knowledge about the pathogenesis underlying this complex disease.

### 4.1. The Association with HLA

Among the identified JIA genetic loci, the human leukocyte antigen locus (*HLA*) confers the strongest effect in JIA genetic susceptibility because of its major roles in autoimmune destruction (Table 2) [43]. The *HLA* genes locate at Chr6p21.3, including *HLA class I* and *class II* genes. Antigens presented by *HLA class I* molecules are of intracellular origin, while antigens presented by *HLA class II* molecules are from extracellular proteins. The HLA proteins are by far the most polymorphic products encoded by the human genome. Multiple amino acid variations have been driven by the evolutionary advantage of heterozygosity, allowing the presentation of diverse antigens from emerging pathogens, despite increased risk of many polygenic diseases [44].

#### 4.1.1. Oligoarthritis and Polyarthritis RF-Negative

In the past few decades, studies have provided multiple lines of evidence that polyarthritis RF-negative subtype has a strong association with *HLA* Class II alleles DRB1*08 and DPB1*03, and *HLA* Class I allele *A2* [45,46]. Oligoarthritis is also associated with DPB1*02, DQB1*04, DRB1*01, DRB1*08, DRB1*11, and DRB1*13 [47,48]. DRB1*11:03/11:04 and DRB1*08:01 confer susceptibility to both oligoarthritis and polyarthritis RF-negative JIA patients [49]. Interestingly, DRB1*04 and DRB1*07 confer a very strong protective effect to oligoarthritis and DRB1*15:01 is protective against both oligoarthritis and polyarthritis RF-negative [49,50]. Fine-mapping the *HLA* locus in JIA reveals that extended oligoarthritis and the RF-negative polyarthritis are genetically similar in their HLA associations [51]. *HLA-DRB1* amino acid position 13 showed the strongest connection with oligoarthritis and both RF-positive and RF-negative polyarthritis [51].

#### 4.1.2. Polyarthritis RF-Positive

There have been many earlier studies focusing on the connection between polyarticular RF-positive JIA and *HLA* [52,53,54,55]. Examination of the genotypes of JIA patients by PCR-sequence-specific oligonucleotide probe methodology has shown that HLA-DRB1*04:05 is associated with polyarthritis RF-positive [56]. Within the *HLA* region, the most significant locus is rs3129769. This locus is near *HLA–DRB1*, and in a strong linkage disequilibrium (r^2^ = 0.88) with the SNP rs660895 reported in rheumatoid arthritis (RA) [57,58]. These SNPs map to the HLA–DRB1*0401 allele.

#### 4.1.3. sJIA

As the one of the common subtypes, accounting for 10–20% of all JIA, knowledge on the pathogenesis of sJIA is still lacking. As the patients with sJIA present with prominent systemic symptoms, such as fever, rash, and synovitis, activation of the autoimmune system is suggested. However, any association between autoantibodies and *HLA* in sJIA is still lacking [59]. Research to date shows that *HLA* class II alleles are significantly associated with sJIA [60]. Meta-analysis of six populations with Western European ancestries showed that HLA-DRB1*11 had strong association [60]. The SNP rs151043342 had the strongest association with sJIA among 482 sJIA-associated SNPs in the class II HLA region [60].

#### 4.1.4. Psoriatic and Enthesitis-Related JIA

*HLA-B27* has been discovered in 60~90% enthesitis-related JIA patients [61]. By assessing the prevalence of *HLA-B27* in enthesitis-related JIA and other subtypes of JIA in an Asian-Indian population, the prevalence of *HLA-B27* was highest in the enthesitis-related JIA group (87%) [62]. In 160 patients of South Indian Tamil with enthesitis-related JIA, Kavadichanda et al. found that HLA-B*27 was positive in 109 (68.1%) patients [63]. Among the HLA-B*27 positive patients, HLA-B*27:04 was discovered in 62.5% patients, followed by B*27:05 in 30.2% patients [63]. Similar clinical phenotypes were seen in both HLA-B*27:04 and *27:05 positive patients [63]. Similar results were reported by Shih et al. in a population from Taiwan. Shih et al. conducted a retrospective review in enthesitis-related JIA patients admitted to the National Taiwan University Hospital during 1993–2018. In total, 97% of patients were *HLA-B27*-positive (92% were B*27:04 and 8% were B*27:05) [64]. In contrast, HLA-B*27:05 was more frequent than HLA-B*27:04 in a northern India population with enthesitis-related JIA, whereas HLA-B*27:05 was also suggested as the ancestral and disease-predisposing allele [65].

### 4.2. Association with Non-HLA Loci

To date, many studies have been performed to test the association between JIA and functional candidate genes with known biological functions or associations with other autoimmune diseases, such as rheumatoid arthritis (RA) and type 1 diabetes (T1D). In addition, a number of JIA-associated non-*HLA* loci have been identified through the unbiased approach of genome-wide association studies (GWAS). Most of these loci are located at noncoding regions, about a half of which overlap with adult-onset arthritis [42,66].

Due to the low prevalence of JIA, sample size has been a major limitation for GWASs on JIA. To increase the sample size, several GWASs have been conducted with a cohort of mixed JIA subtypes. In the first GWAS on JIA, 279 cases (including 48% persistent oligoarthritis, 29% extended oligoarthritis, 20% RF-negative polyarthritis, and 3% RF-positive polyarthritis) and 184 controls were studied. Besides the most strongly associated SNP (rs2187684) mapped to the *HLA* region, Hinks et al. identified a significantly associated SNP (rs2358820) located within the gene *VTCN1*, which encodes a negative regulator of T-cell mediated immune responses [67]. By fine-mapping of the *VTCN1* locus, Hinks et al. identified 10 SNPs associated with JIA [67]. Consequently, a GWAS including 388 children with JIA cases and 2500 controls with European ancestry replicated three previously reported loci mapped to the genes *PTPN22*, *IL2RA*, and *ANTXR2*. Besides, they identified a novel locus with the peak SNP (rs953387), an expression quantitative trait locus (eQTL) associated with the expression level of *CXCR4* in T-cells (*p* = 0.0054) and lymphoblastoid cells (*p* = 0.014) [68]. They also observed four rare variants existed only in JIA patients, not in controls.

The protein tyrosine phosphatase non-receptor type 22 gene (*PTPN22*) variants have been identified of association with JIA and many other autoimmune diseases [69]. *PTPN22* encodes a protein tyrosine phosphatase, which is mainly expressed in cytoplasm of hematopoietic origin cells. The proteins PTPN22, SHP-1 (PTPN6), and PTPN2 may limit TCR proximal signaling by dephosphorylation of Lck Y394 (a key tyrosine residues of Src family) in T-cells [70,71]. Previous studies showed that the counts of effector/memory T-cells increased in *Ptpn22* deficient mice, compared with wild-type mice during rest state, but had no influence on naïve T cells [72,73]. Consistently, *PTPN22* expression has been shown elevated in effector and memory T-cells, relative to naïve T-cells [74,75]. *PTPN22* may thus be important for the regulation of effector but not naïve T-cell activation.

**Figure 1:**
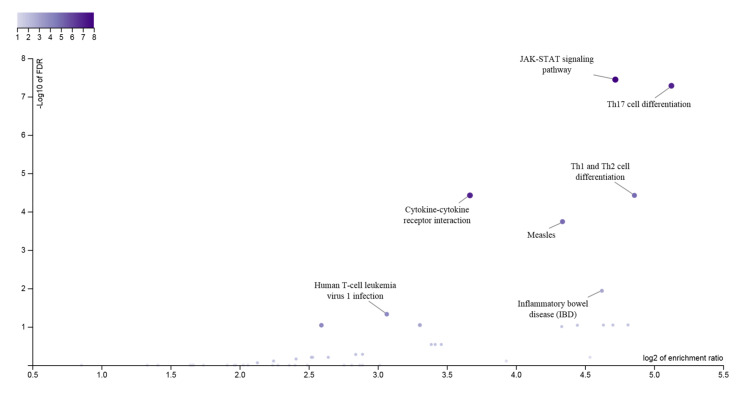
Pathway analysis of 33 genes at the JIA GWAS loci. The enrichment ratio for the functional categories in the KEGG database is shown on the *X*-axis and the log of the FDR is shown on the *Y*-axis, indicating the degree by which the significant categories stand out from the background. The size and color of the dot are proportional to the number of input genes falling into each pathway.

**Figure 2:**
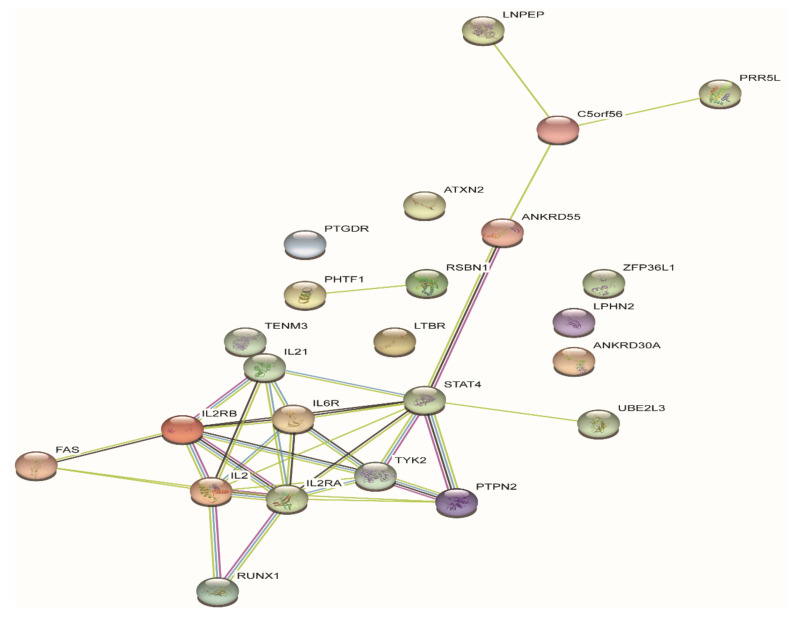
Protein–protein interaction (PPI) analysis of the 33 genes in the GWAS loci. Each node represents each gene product at the JIA GWAS loci and edges between nodes represent protein–protein interactions.

To date, 23 JIA loci, harboring 33 genes, outside of the *HLA* region have been identified with genome-wide significance (Table 3). To systematically examine potential pleiotropic effects of genes associated with different autoimmune diseases, Li et al. conducted a meta-analysis on GWASs of 10 different pediatric-age-of-onset autoimmune diseases [76]. SNPs at seven loci of *ADGRL2*, *PTPN22*, *TENM3*, *ANKRD55*, *IL2RA*, *IL21*, and *ANKRD30A*, were identified of association with both JIA and other autoimmune diseases, such as common variable immunodeficiency (CVID), autoimmune thyroiditis (THY), ankylosing spondylitis (AS), celiac disease (CEL), ulcerative colitis (UC), Crohn’s disease (CD), and systemic lupus erythematosus (SLE), although some loci with opposite direction of effect [76]. The genes at JIA GWAS loci are enriched in signaling pathways playing key roles in both innate immune responses and adaptive immunity, showing interactions with each other (Figure 1 and Figure 2).

#### 4.2.1. Oligoarthritis and Polyarthritis RF Negative

Four previously reported loci of autoimmune diseases, including *PTPN22* (rs6679677, rs2476601, rs2488457), *STAT4* (rs3821236, rs7574865), *C12orf30,* and *ADAD1-IL2-IL21* (rs17388568, rs13143866), and three novel loci including *PTPN2* (rs1893217, rs7234029), *COG6* (rs7993214), and *ANGPT1* (rs1010824), were identified of association with oligoarthritis and polyarthritis RF-negative [77]. This study included 809 oligoarthritis and polyarthritis RF-negative cases and 3535 controls of non-Hispanic European population. Consequently, this group proved the relationship between oligoarthritis and polyarthritis RF-negative by sharing common genetic susceptibility loci, including *C12orf30* (rs17696736), *C3orf1*, and *CD80* at chr3q13 (rs4688011), *JMJD1C* (rs6479891, rs12411988, and rs10995450) [78]. These SNPs have been identified of association with other autoimmune diseases such as RA, T1D, Crohn’s disease, and multiple sclerosis.

In the largest GWAS on JIA to date, Hinks et al. identified 14 novel JIA susceptibility loci with genome-wide significance (*p* < 5 × 10^−8^) by genotyping 2816 JIA cases with European ancestry (including both oligoarthritis and polyarthritis RF-negative patients) and 13,056 controls with the Illumina Infinium Immunochip [42]. The Immunochip provides cost-effective genotyping solution for both common and rare variants as a helpful tool for immunogenetic study. The Immunochip is suitable to conduct replication of autoimmune and inflammatory diseases, and to fine-map established GWAS loci [79]. Another GWAS study on JIA with a large sample size, including 2751 oligoarthritis and polyarthritis RF-negative patients and 15,886 controls, replicated nine previously reported GWAS loci, and suggested five additional loci with *p* < 1 × 10^−6^, i.e., *PRR9_LOR, ILDR1_CD86*, *RNF215*, *LINC00951*, and *HBP1*, but none of these loci reached genome-wide significance [80].

Among the GWAS loci, CD86 and JAK1 are important for signaling pathways of T cell differentiation and proliferation [81]. Many of the oligoarthritis and polyarthritis RF-negative GWAS loci were shared with other autoimmune diseases, suggesting pleiotropic effects of these loci as well as shared molecular mechanisms of autoimmune diseases, such as T-cell receptor activation and signaling. For example, these JIA associated loci have been identified of association with other autoimmune diseases: *JAK1* with CEL and multiple sclerosis (MS); *PTH1R* with CEL; *AHI1_LINC00271* with CEL, MS, THY, and T1D; *WDFY4* with SLE.

Zervou et al. investigated the nonsynonymous SNP rs34536443 on *TYK2* [82], which has been shown of association with various rheumatic diseases. The SNP results in amino acid substitution of Pro1104 to Ala. The study found that the SNP led to the extension of the α-helical segment and alternation of the protein 3D structure, which is likely to affect the function of the TYK2 protein [82]. Couturier et al. performed a study on the association between *TYK2* polymorphism and multiple sclerosis in 1366 French patients and 1802 matched controls [83]. The study found that the protective genotype of the *TYK2* polymorphism rs34536443 may reduce the activity of TYK2, which may promote the secretion of Th2 cytokines and induce T lymphocyte differentiation toward a Th2 phenotype [83]. These functional effects of *TYK2* polymorphism may explain its association with oligoarthritis and polyarthritis RF-negative JIA [42].

#### 4.2.2. Polyarthritis RF-Positive

Some children with polyarthritis RF-positive JIA have the phenotypes of chronic inflammatory arthritis and one or more positive tests of RF and/or anti-citrullinated peptide antibodies (ACPA) [84]. These phenotypes are similar to adult-onset RA. Polyarthritis RF-positive JIA is likely to share clinical features, pathogenesis, and genetic/environmental factors with RA in adults. Previous candidate gene association studies have shown that polyarthritis RF-positive JIA is associated with adult RA-associated loci, i.e., rs10499194 (*TNFAIP3*), rs2476601 (*PTPN22*), and rs7574865 (*STAT4*) [85]. Furthermore, large sample studies on polyarthritis RF-positive provide solid evidence that this infrequent subtype of JIA not only has a lot in common with adult seropositive RA in phenotype, but also has more in common with adult RA in genetics than the other subtypes of JIA.

Hinks et al. recruited 340 polyarthritis RF-positive JIA patients and 14,412 controls from US, UK, Germany, Canada, and Norway, and genotyped these participants using the Immunochip array [57]. This study assessed 44 previously reported non-HLA loci associated with RA, and 27 oligoarthritis/polyarthritis RF-negative JIA loci, to see if these loci were associated with polyarthritis RF-positive JIA in this population. In total, 19 of 44 RA risk loci showed significant association with polyarthritis RF-positive (23 polyarthritis RF-positive associated loci in total) [57,66]. On the other hand, only three of 44 RA risk loci showed significant association with oligoarthritis and polyarthritis RF-negative (27 oligoarthritis/polyarthritis RF-negative associated loci in total) [57,66]. This study provided convincing evidence that polyarthritis RF-positive JIA is more similar to adult RA in genetic architecture than oligoarthritis and polyarthritis RF-negative JIA. In contrast, six loci were shared by polyarthritis RF-positive (out of 23 loci) and oligoarthritis/polyarthritis RF-negative JIA (out of 27 loci) [57].

#### 4.2.3. sJIA

No reported genetic risk factors have been shared by sJIA and other JIA subtypes, suggesting that sJIA may have a special disease process which distinguishes it from other types of JIA. Many candidate gene studies reported sJIA association of a number of SNPs at different genetic loci, such as IL6 [86,87], MIF [88,89], IL10/20 [90,91], IL1 [92,93], MVK, TNFRSF1A [93], CCR5 [94], SLC26A2 [95,96], and TPSN [96], etc. The first GWAS on sJIA included 982 sJIA cases and 431 controls from nine countries [97]. Besides the HLA locus, a novel locus at chr1p36.32 was reported of sJIA association with genome-wide significance (GWS). This locus contains 14 SNPs with GWS, while the index SNP rs72632736 is mapped 263.5 kb upstream of the adherens junction-associated protein 1 gene (AJAP1) [97], which plays a role in cell adhesion and cell migration [98]. The sJIA GWAS also observed 23 novel loci with suggestive evidence (*p* < 5×10^−6^), and found no overlapped genetic loci between sJIA and other common subtypes of JIA [97]. More notably, they found treatment-related susceptibility loci in sJIA, i.e., the *HLA* class II locus. The *HLA* class II molecules may result in activation of CD4+ T cells. Abatacept has shown promising therapeutic effect particularly in refractory cases with sJIA [99,100] by reducing T-cell activation through costimulatory inhibition.

According to the study by Arthur et al. [101], a SNP in the promoter region of *IL1RN* was associated with sJIA. In addition, many of the top sJIA-associated SNPs were correlated with *IL1RN* expression in lymphoblastoid cell lines. It has been observed in sJIA patients that homozygotes of alleles with higher expression of *IL1RN* have strong association with non-response to anakinra therapy. This discovery has important implications in precision treatment of sJIA.

## 5. Transcriptome Study of JIA

Transcriptome study has demonstrated the important roles of specific cell types in JIA pathogenesis of different subtypes, clinical remission, and response to treatment. Differential gene expression analysis uncovered a gradient of order among the JIA subtypes, from healthy controls, to oligoarticular, polyarticular, and sJIA, to Crohn’s disease [102]. Approximately 246 genes were identified as significantly upregulated in patients with active sJIA [103], including IL-6 expressed in monocytes and B cells, IL-10 in monocytes, and suppressor of cytokine signaling 3 (SOCS3) in monocytes and T cells. Cell type analyses allow further probe into the pathogenic mechanisms. Hierarchical clustering suggested the division of polyarticular JIA into three distinct subsets [104]. The three subgroups were associated with different monocyte markers, transforming growth factor β (TGF- β)-inducible genes, and immediate early genes, respectively.

### 5.1. Transcriptome Profiling of Neutrophils in JIA

Ter Haar et al. showed that neutrophils played an important role in the early inflammatory phase of sJIA, and the number and inflammatory activity of neutrophils were highly sensitive to the blockade of IL-1 signaling [105]. To examine activated neutrophil subsets, Brown et al. isolated neutrophils from children with active sJIA or clinically inactive disease (CID) [106]. In both groups, neutrophils from sJIA patients exhibited increased S100A8/A9 release upon PMA stimulation compared to control neutrophils. The study also showed that the number of suppressive neutrophils CD16 + CD62L^lo^ increased with nuclear hypersegmentation in the children with sJIA. Transcriptome analysis of purified neutrophils showed that the genes *AIM2*, *IL18RAP*, and *NLRC4*, involved in critical immune system processes were significantly upregulated [106]. These studies increased our understanding on the roles of neutrophils in sJIA.

### 5.2. Transcriptome Profiling of Macrophages in JIA

CD163, a hallmark of macrophage activation [107], is highly expressed in macrophages of patients with active sJIA or macrophage activation syndrome (MAS) [108]. Two microRNAs, miR-125a-5p and miR-181c, which were previously found to be elevated in active sJIA [109], may reduce CD163 expression of macrophages. Both microRNAs were not elevated in macrophages with stable CD163 expression, but were induced in macrophages with increased CD163 mRNA expression. The miR-181s may target directly at CD163 mRNA for degradation. Overexpression of miR-181c inhibits the anti-inflammatory response of CD163 to hemoglobin or the high-mobility box 1 complex [110]. In contrast, transcriptome analysis of miR-125a-5p overexpression identified “cytokine–cytokine receptor interactions” as the most significantly inhibited gene pathway with reduced interleukin 10 Receptor Subunit Alpha (*IL10RA*), while IL-10 mediation is required for CD163 expression [108].

### 5.3. Transcriptome Profiling of Monocytes in JIA

Comparing JIA patients with controls, CXCL8 (IL-8) was the most significantly expressed gene transcript [111]. The difference in clinical response to methotrexate in patients with JIA was related to the difference in a number of gene transcripts regulated in monocytes [111]. These gene expression profiles could provide the basis for predicting biomarkers of response to methotrexate treatment [111].

### 5.4. Transcriptome Profiling of T Cells in JIA

*CD27, CD276, CTLA4, IL2RG,* and *SLAMF7* are significantly upregulated in patients with sJIA-related lung diseases, suggesting the activation of T cells [112]. By the transcriptome analysis of 579 immune genes of T cells, *UBE2L3*, *IL-6*, *STAT4*, *TYK2*, *TNFAIP3,* and *PTPN2* were significantly dysregulated in the relapse and remission of JIA cases [113]. Upregulation of *HLA* class II and another less-appreciated marker of T-cell activation, CD86, was found in the transcriptome profiling of 92% of JIA samples tested for DNA methylation [114]. Super-enhancers related to autoimmune diseases exist in T cells. Pro-inflammatory signals may inhibit the expression of disease-related genes by inhibiting the activity of super-enhancers [115]. Compared with that in sJIA patients, the number of HLA-DR + T cells and T cells coexpressing CD57 and CD16/56 increased in oligoarthritis JIA and polyarthritis JIA patients [116]. In general, transcriptome analysis of T cells is important for understanding the pathogenesis of JIA as well as its symptomatic treatment.

## 6. Epigenomics Study of JIA

It is well known that only about 2% of sequence in the human genome encodes proteins [117]. What roles do the remaining 98% noncoding regions play? Studies by genome annotations provided evidence that noncoding sequences can actually be transcribed into functional RNA molecules or bind transcription factors to fine-tune gene transcription in the both physiological and pathological processes [118,119]. In the era of GWAS, investigators discovered that the vast majority of SNPs associated with diseases were mapped to noncoding regions of the human genome, including intronic regions and intergenic regions [120].

Jiang et al. studied whether activation histone marks, i.e., enhancers H3K4me1 and H3K27ac, were enriched in the linkage disequilibrium (LD) blocks, containing 22 SNPs reported in the previous GWAS studies on JIA [121]. Chromatin immunoprecipitation (ChIP) followed by sequencing was used in human neutrophils and CD4+ T cells. The study showed that H3K4me1 and/or H3K27ac were enriched in 15 of 22 known JIA-associated loci. In addition, human CD4+ T cells had 18 regions with H3K4me1 and/or H3K27ac histone marks. The study also investigated noncoding RNAs (ncRNAs) within 5 kb of JIA-associated SNPs in human neutrophils, and found ncRNA transcripts in the rs4705862 and rs6894249 loci as determined by chromatin interaction analysis by paired-end tag sequencing (ChIA-PET) [121]. Consequently, the same research group showed that the JIA-associated LD blocks were enriched with H3K27ac and/or H3K4me1 marks, and these blocks bound with transcription factors in neutrophils [122]. In addition, the genes with critical immune functions, including *HLA-DQA1*, *HLA-DQB2*, *TRAF1*, and *IRF1* have long-distance interactions within the LD blocks in human CD4+ T cells as determined by ChIA-PET [122].

The genome-wide analysis by Ellis et al. identified differential DNA methylation of promoter regions in peripheral blood CD4+ T cells from methotrexate-naïve JIA patients, compared to healthy controls, and found that reduced methylation at *IL32* was associated with JIA [123]. Ai et al. collected fibroblast-like synoviocytes (FLS) from four early RA and three JIA patients, and used the previously reported 11 longstanding RA and 11 osteoarthritis as controls, to study DNA methylome signature. The study showed that early RA and JIA clustered with longstanding RA, but were different from osteoarthritis. JIA segregated with the longstanding RA group, and formed a subset in the RA super-group. These findings suggested that the inflammatory injuries in JIA and RA could be caused by abnormal methylation, and levels of methylation are related to the types and stages of disease [124].

Histone deacetylases (HDACs) are acetylation ‘erasers’ that can deacetylate both histone and non-histone proteins. HDACs are involved in cellular signaling, epigenetic regulation, and important in regulating the function of Teff cells and Treg cells [125]. Besides the above mentioned *HLA* class II molecules related sJIA that may be suitable to use abatacept, studies showed the epigenetic effects of *HDAC9* in regulating critical innate immune processes through deacetylation of histone proteins [126,127]. HDAC inhibitors (HDACi) have been extensively studied for the application to the treatment of autoimmune diseases, such as Givinostat, a pan-class I/II HDACi, which is currently being investigated in JIA [128,129], raising the possibility that HDACi represents plausible targeted therapeutic strategies in JIA.

## 7. Conclusions

JIA is a group of diseases, highly heterogeneous in terms of etiology and clinical presentation. Multiomics studies make significant contribution to the understanding of the genetic basis and molecular mechanism of JIA pathogenesis. Similar to other complex human diseases, only a small proportion of JIA familial cases can be attributable to single-gene mutations. For the majority of sporadic JIA cases, the development of the disease is shaped by genetic, epigenetic elements, and environmental factors. Though JIA was classified into seven subtypes, phenotypic overlap was observed between subtypes, suggesting shared genetic/epigenetic basis. The relative low prevalence of JIA makes it hard to acquire enough samples to carry out GWAS on each individual subtype. Proper statistical methods for meta-analysis are needed to identify genetic loci shared by JIA subtypes by taking into account of pleotropic effects and potentially different effect directions between subtypes, as well as small sample sizes. In addition to genetic components, epigenetic modifications also contribute to JIA pathogenesis and development. The shared and distinct epigenetic regulations between JIA subtypes are even less understood, and the interactions between genetic and epigenetic mechanisms warrant further investigation. Integrative analyses of genetic, epigenetic, and transcriptome data are needed to elucidate the full picture of underlying molecular mechanisms of JIA subtypes and to classify JIA cases by molecular markers, and to further facilitate drug development and drug repositioning. New molecular knowledge on different JIA subtypes encourages us to reconsider the JIA classification, but also highlights novel therapeutic targets to develop a cure for the devastating JIA.

## Figures and Tables

**Table 1:** The genes correlated with monogenic forms of juvenile idiopathic arthritis (JIA).

Genes	Causal Mutations (PMID)	Related Subtype of JIA	Functional Evidence (PMID)	Mechanism
LRBA		Oligoarthritis	Lrba^−/−^ mice produce high levels of serum and secretory IgA (28652580).	Defects in peripheral tolerance.
NFIL3	p.M170I (30552177)	systemic JIA	NFIL3 mutations drive elevated IL-1β (30552177)	Sensitizing for arthritis. Development and rewiring the innate immune system for IL-1 overproduction
LACC1	p.Cys284Arg (27881174) p.Ile254Val (27881174) rs3816311 (27098602) p.Arg414Ter (2917096) p.Ile330del (2917096) p.Cys43Tyrfs*6 (30872671)	systemic JIA	TNF levels were increased in LACC1^−/−^ mice. LACC1 transcripts and protein were upregulated by LPS and other TLR ligands in macrophages and dendritic cells (30510070).	Regulating inflammation.
UNCD13	c.117 + 143A>G (29409136) 753 + 3 [G>A] (18240215) 1579 [C>T] R527W (18240215)	systemic JIA	Munc13-4 was highly expressed in differentiated human NK cells and effector CD8+ T lymphocytes. Munc13-4 expression levels were selectively upregulated upon cytotoxic lymphocyte differentiation (24842371).	Disrupting transcription factor binding.

SYS: systemic arthritis; OLG: oligoarthritis; JIA: juvenile idiopathic arthritis.

**Table 2:** HLA alleles associated with JIA subtypes.

Subtype of JIA	Predisposing Allele	Protective Allele
Oligoarthritis and polyarthritis RF-negative	A2, DRB1*01,DRB1*08, DRB1*11, DRB1*13,DPB1*02,DPB1*03, DQB1*04,	DRB1*04, DRB1*07,DRB1*15:01
Polyarthritis RF-positive	DRB1*04:01,DRB1*04:05	
Systemic JIA	HLA-DRB1*11	
Enthesitis-related JIA	B*27:04,B*27:05	

**Table 3:** Genome-wide significant loci (non-HLA) associated with JIA summarized in GWAS Catalog (https://www.ebi.ac.uk/gwas/). JIA associated SNPs listed in GWAS catalog were clumped into independent loci based on the linkage disequilibrium and distance between SNPs.

Index SNPs	Chr	Position	Region	Ref Allele	Risk Allele	Mapped Gene	Risk Allele Frequency	*p*-Value	Odds Ratio	Associated JIA Subtypes	PMID
rs72632736	1	4389144	1p36.32	A	G	*EEF1DP6-LINC01777*		3.00E–09	2.4	SYS	27927641
rs2066363	1	81771892	1p31.1	C	T	*ADGRL2*	0.34	8.00E–11			26301688
rs6679677	1	113761186	1p13.2	C	A	*PHTF1-RSBN1*	0.10	3.00E–25	1.59	OLG,PRFN	23603761
rs6679677	1	113761186	1p13.2	C	A	*PHTF1-RSBN1*	0.09	8.00E–11			26301688
rs72698115	1	154406893	1q21.3	A	C	*IL6R*	0.1	1.00E–08	1.36	OLG,PRFN	23603761
rs10174238	2	191108308	2q32.3	G	A	*STAT4*	0.23	1.00E–13	1.29	OLG,PRFN	23603761
rs1479924	4	122466445	4q27	G	A	*IL2-IL21*	0.71	6.00E–11	1.27	OLG,PRFN	23603761
rs62324212	4	122639784	4q27	C	A/G	*IL21-AS1*	0.42	3.00E–08			26301688
rs7660520	4	182824168	4q35.1	G	A/C	*TENM3-AC114798.1*	0.26	8.00E–11			26301688
rs10213692	5	56146422	5q11.2	T	C/T	*ANKRD55*	0.75	3.00E–11	1.27	OLG,PRFN	23603761
rs7731626	5	56148856	5q11.2	G	A	*ANKRD55*	0.39	1.00E–10			26301688
rs27293	5	97021474	5q15	A	G/T	*LNPEP*	0.44	7.00E–09	1.31	OLG,PRFN	23603761
rs6894249	5	132461855	5q31.1	A	G	*AC116366.3, C5orf56*	0.61	1.00E–09	1.32	OLG,PRFN	23603761
rs6946509	7	22769871	7p15.3	T	A/C	*MTCYBP42-AC073072.2*	0.45	3.00E–08	1.19	OLG,PRFN	23603761
rs7909519	10	6047878	10p15.1	T	G	*IL2RA*	0.89	8.00E–10	1.39	OLG,PRFN	23603761
rs706778	10	6056986	10p15.1	C	T	*IL2RA*	0.41	6.00E–09			26301688
rs7100025	10	37303610	10p11.21	G	A	*LINC00993, ANKRD30A*	0.34	8.00E–11			26301688
rs7069750	10	89002619	10q23.31	G	C/T	*FAS*	0.44	3.00E–08	1.18	OLG,PRFN	23603761
rs7127214	11	36322143	11p13	C	G/T	*PRR5L, AC087277.1*	0.65	2.00E–08	1.28	OLG,PRFN	23603761
rs10849448	12	6384185	12p13.31	A	G	*LTBR*	0.24	5.00E–09	1.24	OLG,PRFN	23603761
rs7137828	12	111494996	12q24.12	C	A/T	*ATXN2*	0.49	2.00E–09	1.20	OLG,PRFN	23603761
rs3825568	14	68793871	14q24.1	C	G/T	*ZFP36L1*	0.56	1.00E–08	1.30	OLG,PRFN	23603761
rs2847293	18	12782449	18p11.21	A	G/T	*AP005482.1-PTPN2*	0.17	1.00E–12	1.31	OLG,PRFN	23603761
rs34536443	19	10352442	19p13.2	G	C	*TYK2*	0.95	1.00E–10	1.79	OLG,PRFN	23603761
rs8129030	21	35340290	21q22.12	T	A/G	*RUNX1*	0.63	5.00E–09	1.28	OLG,PRFN	23603761
rs2266959	22	21568615	22q11.21	G	T	*UBE2L3*	0.19	6.00E–09	1.24	OLG,PRFN	23603761
rs2284033	22	37137994	22q12.3	G	A	*IL2RB*	0.56	2.00E–08	1.19	OLG,PRFN	23603761

PRFN, polyarthritis RF negative; OLG, oligoarthritis; SYS, systemic arthritis; JIA, juvenile idiopathic arthritis.
